# A Comparative Study on Acceptance and Distribution of Modern Medical Care in Japanese Colonies

**Published:** 2018-07

**Authors:** Do-Hyung KIM, Eun-Young PARK

**Affiliations:** Academy of East Asian Studies, Sungkyunkwan University, Seoul, South Korea

**Keywords:** Modern medical care, Civilization, Japanese colonies, Traditional medicine

## Abstract

**Background:**

The propagation of modern medicine in the colonies has often been described in terms of modernism and nationalism, focusing on the action-reaction conflict with colonial power, but the propagation of modem medical care and hygiene in colonial Chosun seems not to be explained by this perspective. So how can we explain this aspect? Answering this question could provide new implications for the many controversies surrounding the “colonial modern” acceptance.

**Methods:**

In order to examine the pattern of colonial acceptance of modern medicine, three cases of Britain and India, Japan and Taiwan, Japan and Chosun were set and compared, and the characteristics of colony Chosun were examined in-depth.

**Results:**

The existence of the ‘traditional medicine doctors’ who played an important role in the dissemination of public health in colonial Chosun can be explained from the understanding of the identity of Confucian intellectuals who played a role in the traditional Chosun society.

**Conclusion:**

The proliferation of modern medicine in the colonies has often been explained in terms of modernism and nationalism; however, the acceptance of a modern sanitary system in colony Chosun has been influenced by the traditional elements of the society. When considering these factors, the relationship between colonialists and the colonized society can be examined from a more interrelated perspective.

## Introduction

The prevalence of pests and cholera that raged in the nineteenth century validated the superiority of modern Western medicine and influenced the establishment of the quarantine systems and infrastructures of sanitation. Japan promptly imported Western medicine with the intention of becoming a modernized country. Modern Western medicine was understood contemporarily as a symbol of modernity and was used to distinguish countries and their people as either ‘civilized’ or ‘barbarian’, depending on the presence or absence of modern medicine and sanitation systems. Traditional medicine, on the other hand, was regarded as a symbol of uncivilized inheritance.

In 1899, Japan restored the authority of quarantine simultaneously with the abolishment of consular jurisdiction and foreign concession (1). This can be interpreted as Japan achieving the state of ‘being civilized’ out of the state of being ‘barbarian’ and ‘unsanitary’, through the introduction of a modern system of ‘sanitation’. The modern medical care system was established in Japan promptly, gradually replacing traditional medicine. The replacement was regarded as evidence of a more modern civilization. Modernity of the Japanese medical care system and policies established for the propagation thereof gained momentum to distinguish the status of the Japanese empire from its peripheral countries; the system and policies were recognized as important mechanisms to cement the legitimacy of its domination.

As it had been before, in Taiwan, the first country colonized by the contemporary Japanese empire, the Western medical systems and policies, established for modern sanitation systems, were implemented broadly as a symbolization of modern civilization. The implementation of such policies propagated to other regions under colonial governance, such as Kwantung Leased Territory of China, Chosun, and the South Pacific Islands (2–5). The modern Western medicine and sanitation projects propagated by Japanese colonial power were often recognized as aspects of ‘beneficial governance’. These were sometimes used in studies that emphasized the (beneficial) effects of such sanitation policies over the colonial governance. However, in other studies, the policies of sanitization were seen as a suppressive administration of colonial power, and sought confrontation with or resistance against Japanese imperial colonialism. Other studies concluded the establishment and implementation of policies for the modern medical care system in Chosun was relatively inferior to that of Taiwan, despite the introduction of most policies established and implemented in Taiwan to Chosun as a strategic means to rule Chosun colony (6). Additionally, some studies found that the differences in the establishment and implementation of such policies between the two different colonies, ascribable to the values of the two disparate colonies, were recognized differently by Japanese colonialists (7). Such studies are based on the monolithic view, and commonly took the subjects under colonialism in their studies as an action of, or reaction against, colonial power.

This study aimed to objectively observe the interaction of the colonized societies with the colonial power.

## Methods

### Case of India

Nonintervention of Colonialists in Colonial Society ↔ Intensification of an Initiative on Colonial Society

### Case of Taiwan

Active Engagement of Colonialists in Colonial Society ↔ Attenuation of an Initiative on Colonial Society

### Case of Chosun

Passive Engagement of Colonialists in Colonial Society ↔ Securing of an Initiative on Colonial Society

The non-intervention of a colonialist in his colony would intensify its initiative on colonial society, whereas, the intervention of colonialist in his colony may attenuate or reduce its initiative on the colonial society. Here, the judgment on the degree of intervention of colonialist does not necessarily correspond to the emphasis of unidirectional influence of colonialist, but instead, on the assumption of the reaction of the colonials dependent on the reaction of colonized societies, the explorations conducted in this study focused on the interaction between the colonialist and the colonials and the reasons behind such interaction. This would enable the study to not only consider the effect and variable of the degree to which the colonizer intervened in a colony with regard to modern medical care’s establishment but also identify the effect and significance of each colony’s own uniqueness (tradition) on the colonizer’s policy establishment and propagation.

## Results

### Case of Colony India

The British pursued rapid colonization in Bengal, India since the late eighteenth century and made protecting themselves from the region’s tropical climate and conditions one of their most important tasks. In other words, Britain had a “hygiene” policy in India for British people stationed in India, which was characterized by non-intervention in local India. To the British, India’s tropical climate meant unpleasant and unhealthy conditions that resulted in a high mortality rate. Cholera, which broke out repeatedly in the nineteenth century, was perceived as originating from Indians’ unhygienic habits and lifestyle. British people’s strong intolerance for India’s climate and dissimilarities with local Indians further increased their distance from local India. In fact, the separation of residential districts for the ruling British and the ones for those who were ruled was a typical spatial arrangement found in cities across colonial India. Moreover, the massive hygiene reforms, which were introduced after the Indian Rebellion of 1857, were also made only for British residential districts (1).

A plague spread across many parts of India in the late nineteenth century and claimed many lives. The spread of the plague served as an opportunity to remind people of the importance of Britain’s hygiene intervention in India, but it was insignificant, as it faced strong opposition from India. Nevertheless, Britain’s hygiene policy characteristics led to the following phenomena in India:

First, as the colonizers distanced themselves more from local India, the middle class’s interest in Western medicine grew even further in colonial India. An emerging class from high castes learned Western medicine, opened their businesses, and practiced medicine in the private sector. Furthermore, it is interesting that they emerged not only in medicine, but also in politics. They participated in various political organizations in urban and rural areas, and were involved greatly in the development of Indian nationalism. Second, the dominance of Western medicine had been proven across the world throughout the nineteenth century. In the late nineteenth century, when the absolute dominance of Western medicine was clearly established, a movement to revive Ayurveda, traditional medicine, began and resulted in different types of actions, such as establishing medical schools to nurture traditions, and holding qualification tests for traditions. Overwhelming support for traditional medicine from a majority of people in India who had never encountered Western medicine was a natural phenomenon, and the emergence of a nationalist movement at that time also had a huge effect on the revival of traditional medicine (1).

Nonetheless, the two seemingly conflicting phenomena—vigorous interest in Western medicine on one hand, and the revival of traditional medicine on the other hand—could be viewed as local India’s strengthened initiative in response to the colonizer’s non-intervention in local India. Although these two phenomena were not necessarily favorable to one another in many cases, both were the result of the colonial country taking the initiative against the colonizer.

### Case of Colony Taiwan

Japan proactively sought to establish a modern medical care system in Taiwan, its first colony. In early 1874, most Japanese soldiers were infected with malaria when they were dispatched to Taiwan, and it had continued through their occupation of Taiwan in 1895. Above all, since deaths due to malaria and plague in Taiwan could have also had a huge effect on Taiwan’s demographics, it was a top priority to curb the spread of infectious diseases to make things easier for Japan’s colonial rule.

The role of Goto Shinpei, who was appointed as Civil Administrator, was important for the Governor-General of Taiwan to implement various policies in Taiwan based on Japan’s domestic hygiene administrative measures. Despite Taiwan’s backlash, Shinpei reorganized the traditional baojia system, organized hygiene associations based on the system, and actively implemented epidemic prevention measures (5). Yanaihara Tadao, who left behind systemic research on Japan’s colonial rule in Taiwan, stated that the implementation of such a hygiene project not only reduced “evil diseases” such as plague and malaria, and made it easier for Japanese people to come and live in Taiwan, but also improved the local Taiwanese people’s hygiene significantly. He also assessed the accomplishments of the Governor-General’s hygiene project as commendable (8). This “systematization” of hygiene helped the colonizer to build a relationship with the local country and inject colonial power deeper into the country. Compared to the aforementioned case of India, it is notable that Japan is characterized by its use of an aggressive intervention, thorough the development of a hygiene project in the local country.

Accordingly, the hygiene police were quite powerful in colonial Taiwan. The hygiene police system in Taiwan was characterized as being centralized and militaristic. It controlled both administrative rights and police rights, and was largely affected by Japan’s national and military medicine. In general, when the colonizer’s national medicine was applied to a colony, a hygiene project showed that the local country’s hygiene had not yet been “civilized,” mostly based on the colonizer’s view of hygiene. This led to the logic that the colony should be saved by the superior colonizer; in this regard, it was an important tool used to destroy the colony’s tradition and pride. This enabled the colonizer to take control of the colony’s medical and hygiene system, and allow the hygiene police to oversee the hygiene administrative works using coercion based on the criteria set by the government (9).

There was no room for Han doctors or Chinese doctors, who practiced traditional medicine in Taiwan, to do something amid the colonizer’s strong expansion of Western medicine and hygiene projects. Since the first examination to give a license to traditional doctors was held in 1901 based on Taiwanese doctor license rules, there had been no other examination, and the number of traditional doctors decreased from 1,223 in 1901 to 97 in 1942; they were essentially wiped out. With the colonial government’s measures to exclude traditional doctors, the Governor-General established medical schools and attempted to nurture doctors who could perform Western medicine. In establishing and operating medical educational institutions to nurture doctors, Japan took a different approach in Taiwan from that in its home country. In short, medical schools established in Taiwan emphasized the education of clinical medicine needed for the colonizer’s rule, and nurturing and increasing the number of highly practical practitioners were medical education’s key goal and a part of the colonial rule (9).

As demonstrated above, in establishing a modern medical care system in Taiwan, Japan increased the influence of its colonial power via the thorough “systematization” of hygiene, and controlled the colony’s medical and hygiene system through the hygiene police system. During this process, Taiwan’s traditional medicine lost its role and basically became extinct (10). The colonizer’s aggressive intervention in the local country significantly weakened the colony’s initiative, and Taiwan’s traditional medicine did not gain any status under the colonizer’s planning and control; the colonizer’s needs were accepted as the colony’s needs.

### Case of Colony Chosun

As demonstrated in the above mentioned two cases, it is worth noting that India and Taiwan had significantly different climatic conditions from their colonizer’s home country. Given that both Britain and Japan needed to maintain their “rule” in their colony, the health of the managing rulers and their military was a top priority. In other words, Britain in India and Japan in Taiwan both faced a challenge of controlling the climate and infectious diseases that they had never experienced before. However, given that it was difficult to find such climatic differences in the case of Chosun, Japan expected to experience similar things that it encountered when it first introduced modern medical care in its home country.

During the period of the Japanese empire as a protectorate (1905–1910), Japanese colonial power did not immediately prohibit the practices of traditional medicine or the practices of doctors of traditional herbal medicine. The colonial powers allowed them to practice freely and be gradually phased out over time, despite their intention to transplant the modern system of Western medical care. Ito Hirobumi, the governor of protectorate Chosun, recognized that since the medical care system would be reorganized naturally around Western medicine with the propagation of modern civilization, it would not be necessary to rush it and invite an unnecessary backlash (11). These were based on previous experiences of the successful implementation of the modern system of Western medical care, completed without the permission of reproduction (education) and sustainment (licensing) of the doctors of traditional medicine in Japan. This continued after Japan’s annexation of Chosun in 1910. During the colonial rule, those who mostly provided medical care in the private sector in Chosun were traditional medicine doctors and herbal medicine doctors (12). Above all, a difference between Japan and Chosun was that while Japan mainly practiced Western medicine, it incorporated existing traditional medicine practitioners into the doctor system, and severed the reproduction of those people. In Chosun, a traditional medicine doctor system was put in place separately and traditional medicine practitioners were incorporated into the system as lower-class medical practitioners below doctors. This trend also existed in Taiwan to some extent, so it is fully possible to indicate discriminatory trends between Japan and Taiwan-Chosun, the colonizer and colonies. Many existing studies have pointed to the ‘differentiation’ of colonial power from this point of view (12, 13).

In 1913, with the promulgation of the “Regulations on Doctor · Regulations on Dentists Regulations on Doctors of Traditional Medicine” the Japanese colonial government of Chosun revealed its healthcare plan for the Chosun colony. According to the “Regulations on Doctors of Traditional Medicine”, the doctors of traditional medicine, the herbalists, became the regular medical staff instead of the people formerly providing patients with traditional medical care (14). However, the doctors of traditional medicine, which were supposed to have restricted responsibilities, were discriminated against and differentiated from the doctors licensed with Western medicine, by having their statuses demoted. As in the case implemented in Taiwan, the employment of the doctors of traditional medicine in Chosun, implemented by the Japanese colonial government, was a temporary measure. It was implemented with the anticipation of the eventual extinction of traditional medicine by the strong power of modern Western medicine, as it was already happening in Japan.

However, contrary to the expectations of the Japanese colonial government, the doctors of traditional medicine did not disappear, and the official examinations remained until the end of colonialism, in contrast to the sole case examined in Taiwan. Instead, the number of doctors of traditional medicine increased to 6,000, and they participated in several non-therapeutic or clerical responsibilities such as issuing medical certificates, preventing epidemics, and issuing death certificates etc., as well as in caring for patients (13). They even learned the knowledge of Western medicine spontaneously, applied the knowledge to their medical activities, and established various associations of doctors of traditional medicine (15). Despite the intention to reduce the number of doctors of traditional medicine gradually, the Japanese colonial government relied upon these doctors of traditional medicine, and the colonial government established the training school for the doctors of traditional medicine to teach them basic Western medicine. Doctors of traditional medicine were originally alienated from the domain of the modern medical system but, they were participating in an important part of hygienic administration (16).

## Discussion

As demonstrated above, the colonial power had a very complex relationship with traditional medicine practitioners in Chosun, and it can be easily assumed that this relationship was more complex particularly in rural areas, which were quite far away from cities in terms of time and space. Among others, there were Confucian Doctors who called themselves Confucian scholars and practiced medicine, and they had an ambition of realizing Confucian ideals by linking them with medicine in traditional society.

First, it is necessary to describe the notion of “Confucian doctors.” Confucian doctors are not defined as a system or social class, but instead, they are a sort of a symbol or self-defined notion. Confucianism is represented by the saying that one should cultivate himself, then regulate the family, then govern the state, and finally lead the world into peace, it is an ideological system that assumes a unified connection between the human body, the state, and the world. Confucian order could not be unrelated to an individual’s physical body and health, and it was a mandate for medicine to embody Confucian ideals in the East Asian world influenced by Confucianism. For instance, according to studies on those who called themselves Confucian doctors during the Ming and Qing dynasties in China, medicine was considered another pathway for influential figures in rural areas who could not get into the central government through the imperial examination since the Song dynasty; practicing medicine was also used to show the identity of Confucian intellectuals who embodied the world order in rural areas. They were mostly a family of doctors and continued to take the imperial examination. Even though their ultimate goal was to serve the country as state officials, it was not rare that they practiced medicine if it did not go well. They built medical virtues based on Confucian criteria and the values that regulated them, and established various ethical criteria accordingly; they established and evaluated their position in the world order explained by Neo-Confucianism, and they served as those who connected the central government and local communities, and maintained the order underpinned by Confucianism and its rules, even though they were not state officials (17–19).

A recent study on Kim Kwang-jin, who called himself a Confucian doctor during the colonial period in Chosun, is quite noteworthy (20). He lived his entire life identifying himself as a Confucian and worked as a doctor. Kim, who was born in 1885, became interested in medicine in 1915, when he was not very young. The reason he wanted to become a doctor was that he wanted to treat his father’s disease. This was a typical reason for someone with a Confucian identity to be interested in medicine (17). Even though studying Confucianism was not as significant as it had been since the state official examination was abolished in 1895, there would have been a countless number of people who did not give up the identity of a Confucian, whose life goal was to become a state official, serve the country, and nurture future generations. Kim was one such figure, and his participation in the March 1 Movement, and his service as a principal in a private school called Duksan are considered to demonstrate the activities of a typical Confucian in this period (16).

It was in 1924 when Kim was around 44 years old that he took the examination and became a doctor of traditional medicine. Interestingly, Kim picked Fan Zhongyan (989–1052), one of the greatest statesmen in the Song dynasty, as a figure he admired throughout his life (16). Fan was known for a resolution he made in a temple before he became a state official that “if I cannot become a prime minister in a peaceful world and serve the world, I will become a good doctor and treat people’s diseases.” Becoming someone like Fan was another path through which Kim could practice the ideal of “save the world and people,” which he could not fully realize due to the limitations of the historical period he was in. Even though Fan did not become a doctor, he was recognized as a figure who best represented the ideology that medicine and Confucianism were identical in terms of practicing “benevolence” (18) and it was natural that many doctors not only in the pre-modern period but also in the colonial period admired him as a role model (16).

As demonstrated above, medical practitioners in the Chosun period and “traditional medicine doctors” during the colonial period did not aim to become doctors and progress in their careers or advance their personal gains by treating patients, but wanted to define their lives in terms of the traditional Confucian ideology and live as individuals who served as a part of the order connecting the rural areas to the world. Even though they did not become “yangbans” through the state official examination, it is important to understand the activities of these traditional medicine doctors as people who practiced Confucian values in rural areas.

The cases of Chosun were different from those in India, Taiwan, and in Japan. Most of the previous studies presented interpretations excessively oriented towards modernity or nationalism. But considering the actual realities of the doctors of traditional medicine in Chosun that had defined their lives and shared their identities resulting from the definitions made under the strict order of Confucianism, the mode of existence of the ‘doctors of traditional medicine’ after the period of colonialism, can be possibly understood in new perspectives. The mode of existence of the doctors of traditional medicine in colony chosun can be perceived as a new transformation of traditional order, rather than an existence, to be interpreted as national resistance against or compliance with colonial power, or simply as a matter of superiority or inferiority in the orientation towards modernity.

## Conclusion

The situation of healthcare in colony Chosun, sustained by the presence of such doctors of traditional medicine, can be regarded as one of the specialized particularities. The policies of modern medical care implemented by the Japanese colonial government in Chosun are different from those implemented in Taiwan, possibly due to the discrimination between colonies, as it was pointed out by prior studies. Otherwise, the discrimination may have been selected as a strategy for exploiting traditional systems and human resources in order to accomplish an economy of high efficiency with low cost, without the use of an active intervention. It would be reasonable to assume that the people engaged in traditional medical care had found their own initiatives in their own space (of the officially authorized occupation as doctors of traditional medicine) prepared in the system of modern medical care, and thereby, actively accustomed themselves to the space. The limited or lack of human resources for medical care based on Confucian identity in Taiwan or in Japan may have also been a determining factor for such differences.

In this context, the aforementioned third type, the type that assumed the ‘Passive Engagement of Colonialist in Colonial Society ↔ Securing of an Initiative on Colonial Society (the case of Chosun)’, can be regarded as a type showing the unique colonial characteristics of Chosun contrasting to other societies.

## Ethical considerations

Ethical issues (Including plagiarism, informed consent, misconduct, data fabrication and/or falsification, double publication and/or submission, redundancy, etc.) have been completely observed by the authors.
